# Recent Progress in the Management of Retroperitoneal Sarcoma

**DOI:** 10.1080/13577140120048908

**Published:** 2001-03

**Authors:** Rona Cheifetz, Charles N. Catton, Rita Kandel, Brian O'Sullivan, Jean Couture, Carol J. Swallow

**Affiliations:** 1 Department of Surgical Oncology Mount Sinai Hospital and Princess Margaret Hospital University of Toronto Ontario Toronto Canada; 2 Department of Radiation Oncology Mount Sinai Hospital and Princess Margaret Hospital University of Toronto Ontario Toronto Canada; 3 Department of Pathology Mount Sinai Hospital and Princess Margaret Hospital University of Toronto Ontario Toronto Canada; 4 600 University Avenue,#1224 Ont. Toronto M5G 1X5 Canada

## Abstract

Retroperitoneal sarcomas (RPS) are rare tumours that typically present late and carry a poor prognosis even following grossly complete resection. In an attempt to improve the outlook for patients with RPS, sarcoma specialists have employed various adjuvant therapies, including extermal beam radiation, intraoperative radiation, brachyradiation and systemic chemotherapy. This article reviews the presentation and prognosis of RPS, and focuses on the results of new treatment strategies compared with conventional management.

A Medline search of the English literature was performed to identify all retrospective and prospective reports relating to the management of adult RPS published since 1980. Series that did not analyse RPS separately from other intra-abdominal or extra-abdominal sarcomas or other malignancies were excluded, and information on investigation, presentation, prognostic factors, treatment and outcome was extracted from the remaining reports. Survival and local control data were collected from reports that contained at least 30 cases of RPS (*n* = 31).

While surgical resection remains the cornerstone of treatment for RPS, the majority of patients will relapse and die from sarcoma within 5 years of resection. Adjuvant radiation may improve these results, but further trials are required to definitively demonstrate its benefit. Possible reasons for the failure of conventional treatment are discussed, and alternative strategies designed to overcome these obstacles are presented.


*Sarcoma* (2001) **5**, 17–26

ORIGINAL ARTICLE

### Recent progress in the management of retroperitoneal sarcoma

RONA CHEIFETZ,[1] CHARLES N. CATTON,[2] RITA KANDEL,[3] BRIAN O'SULLIVAN,[2] JEAN COUTURE[1] & CAROL J. SWALLOW[1]

*[1]Department of Surgical Oncology, [2]Department of Radiation Oncology, and [3]Department of Pathology, Mount Sinai Hospital and Princess Margaret Hospital, University of Toronto, Toronto, Ontario, Canada*

**Abstract**
Retroperitoneal sarcomas (RPS) are rare tumours that typically present late and carry a poor prognosis even following grossly complete resection. In an attempt to improve the outlook for patients with RPS, sarcoma specialists have employed various adjuvant therapies, including extermal beam radiation, intraoperative radiation, brachyradiation and systemic chemotherapy. This article reviews the presentation and prognosis of RPS, and focuses on the results of new treatment strategies compared with conventional management.
A Medline search of the English literature was performed to identify all retrospective and prospective reports relating to the management of adult RPS published since 1980. Series that did not analyse RPS separately from other intra-abdominal or extra-abdominal sarcomas or other malignancies were excluded, and information on investigation, presentation, prognostic factors, treatment and outcome was extracted from the remaining reports. Survival and local control data were collected from reports that contained at least 30 cases of RPS ($n$ = 31).
While surgical resection remains the cornerstone of treatment for RPS, the majority of patients will relapse and die from sarcoma within 5 years of resection. Adjuvant radiation may improve these results, but further trials are required to definitively demonstrate its benefit. Possible reasons for the failure of conventional treatment are discussed, and alternative strategies designed to overcome these obstacles are presented.

### Introduction

Adult soft tissue sarcoma presents in five principal sites, with the retroperitoneum being the least common (Fig. 1). While advances in the local management of extremity sarcoma with combined surgery and radiotherapy have improved long-term local control rates from less than 80% in 1980 to as high as 95% presently,[1,2] the local failure rate for retroperitoneal sarcoma (RPS) remains high. The effectiveness of both surgery and radiotherapy is compromised by the tendency of retroperitoneal tumours to grow silently until they involve adjacent critical and sensitive structures. Predictably, the local control and survival rates for RPS are much worse than for sarcomas arising at other sites (Fig. 2).

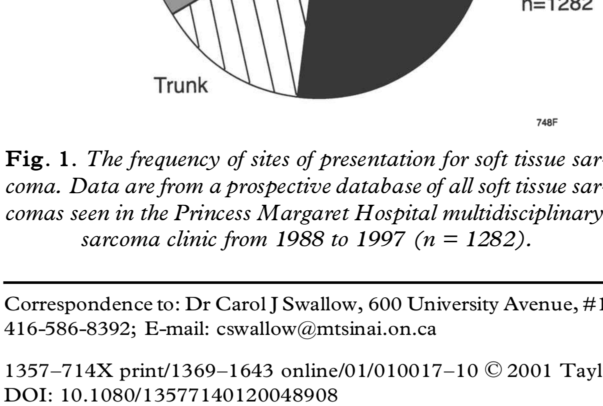

**Fig. 1.** *The frequency of sites of presentation for soft tissue sarcoma. Data are from a prospective database of all soft tissue sarcomas seen in the Princess Margaret Hospital multidisciplinary sarcoma clinic from 1988 to 1997 (n = 1282).*

In most modern series, fewer than 70% of RPS are resected with curative intent at presentation, and at least one-half of the patients who have a grossly complete resection develop a local recurrence (Table 1). The majority of deaths in patients with RPS are due to complications of uncontrolled intra-abdominal disease, rather than to distant metastatic disease.[3–5] This suggests that strategies to improve local control could reduce disease-related morbidity, improve disease-free survival, and possibly improve the cure rate. Since complete gross resection is the only treatment factor definitively shown to improve survival in RPS,[3,4,6–9] several authors have advocated more aggressive *en bloc* resection of the tumour together with adherent organs and structures. The effectiveness of such a surgical

Correspondence to: Dr Carol J Swallow, 600 University Avenue, #1224, Toronto, Ont., Canada M5G 1X5. Tel: (+1) 416-586-1558; Fax: (+1) 416-586-8392; E-mail: cswallow@mtsinai.on.ca

1357–714X print/1369–1643 online/01/010017-10 © 2001 Taylor & Francis Ltd
DOI: 10.1080/13577140120048908

**Table I**. *Treatment outcome for retroperitoneal sarcoma*[a]

| Reference | n | Complete resection rate[b] (%) | Overall Survival[c] 5 year (%) | 10 year (%) | Local control after complete resection[d] (%) |
|---|---|---|---|---|---|
| Petersen *et al.*[e] (in press)[44] | 87 | nr | 47 | nr | 58 |
| Alektiar *et al.*[e] (2000)[33] | 32 | nr | 45 | nr | 63 |
| Eroğlu *et al.*[f] (1999)[24] | 40 | 85 | 49[g] | nr | 45 @ mean 57 months |
| Herman *et al.* (1999)[49] | 70 | 73 | 53[g] | 40[g] | nr |
| Malerba *et al.*[h] (1999)[50] | 42 | 60 | 48[g] | nr | nr |
| Lewis *et al.*[i] (1998)[4] | 500 | 62 | 54[h] | 35[h] | 59[h] |
| Wang *et al.*[h] (1996)[25] | 40 | 70 | 25 | 21 | nr |
| Jenkins *et al.* (1996)[26] | 119 | 49 | 20 | nr | nr |
| Karakousis *et al.* (1995)[21] | 90 | 96 | 63 | 46 | 50 @ min. 5 years, 40 @ min. 10 years |
| Kilkenny *et al.*[h] (1996)[27] | 63 | 78 | 48 | 37 | nr |
| Singer *et al.* (1995)[28] | 83 | nr | 60 | 50 | nr |
| Wang *et al.*[j] (1994)[32] | 30 | 60 | 14 | nr | nr |
| van Doorn *et al.* (1994)[51] | 34 | 88 | 35[g] | nr | 37 |
| Catton *et al.* (1994)[19] | 104 | 43 | 36 | 14 | 50 @ 5 years, 18 @ 10 years |
| Sindelar *et al.*[k] (1993)[35] | 35 | 80 | 45[g] | nr | 37 @ 8 years |
| Shiloni *et al.*[l] (1993)[52] | 41 | 56 | 45 | 33 | 10 @ 10 years |
| Zornig *et al.* (1992)[53] | 51 | 59 | 35 | 15 | nr |
| Alvarenga *et al.* (1991)[3] | 120 | 30 | 29 | nr | 15 |
| Dalton *et al.* (1989)[7] | 116 | 54 | 40 | 22 | 41 @ mean 4.1 years |
| Pinson *et al.*[h] (1989)[54] | 79 | 48 | 44 | nr | nr |
| Bolin *et al.* (1988)[16] | 32 | 62 | 28 | nr | nr |
| Salvadori *et al.*[h] (1986)[55] | 43 | 42 | 11 | nr | nr |
| Karakousis *et al.* (1985)[56] | 68 | 40 | 34 | 22 | nr |
| Glenn *et al.* (1985)[10] | 37 | 72 | 23[g] | nr | nr |
| Wist *et al.*[h] (1985)[57] | 36 | 43 | 22 | nr | nr |
| McGrath *et al.*[h] (1984)[9] | 47 | 38[m] | 32 | 19 | 44 @ median 5 years |
| Shmookler & Lauer[n] (1983)[58] | 36 | nr | 34 | 27 | nr |
| Stower & Hardcastle[h] (1982)[59] | 32 | 35 | nr | nr | nr |
| Cody *et al.*[o] (1981)[20] | 80 | 66 | 45[g] | nr | 23 |
| Storm *et al.* (1981)[5] | 54 | 61 | 33 | 10 | 28 |
| Fortner *et al.*[p] (1981)[60] | 78 | 53 | 37[g] | nr | nr |

nr, Not reported.

[a] Series of 30 or more patients published in English since 1980. All series are retrospective except for Petersen *et al.*, Alektiar *et al.*, Lewis *et al.*, Jenkins *et al.*, Sindelar *et al.*, and Glenn *et al.* Where the same centre has published sequential series that include patients all included in a previous series, only the most recent qualifying publication is quoted.

[b] The proportion of patients with retroperitoneal sarcoma undergoing surgical exploration at that centre who had a grossly complete resection, irrespective of the microscopic margin status. The denominator used to calculate this proportion does not always correspond to $n$, the number of patients included in the series.

[c] Irrespective of resection status, unless otherwise noted. Actuarial except for Sindelar *et al.*, where the survival rate is actual.

[d] Locoregional control rate in patients undergoing complete resection, with or without the use of adjuvant radiotherapy or chemotherapy. Unless otherwise indicated, the figure quoted is actuarial and at 5 years post-resection.

[e] All patients received intraoperative radiotherapy; the majority also received external beam radiotherapy. For Alektiar *et al.*, survival rate is quoted for 32 patients, 30 of whom had complete resection.

[f] Eleven patients received hyperthermic total abdominal perfusion.

[g] Survival quoted for completely resected patients only.

[h] Limited to patients with primary tumours.

[i] Complete resection rate is quoted for patients with primary or recurrent disease excluding those with distant metastases ($n$ = 397); survival rates quoted are disease-specific survival in all patients with primary disease ($n$ = 278); local control rate quoted is for patients with primary disease undergoing either complete ($n$ = 185) or partial ($n$ = 62) resection.

[j] Limited to patients with locally recurrent disease.

[k] Prospective randomized trial of post-operative radiotherapy with or without intraoperative radiotherapy for completely resected patients; survival and local control rates quoted for both arms combined.

[l] Limited to patients with high-grade tumours.

[m] Complete resection defined as microscopic margins negative.

[n] Limited to patients with leiomyosarcoma.

[o] Rates quoted for patients treated since 1971.

[p] Included eight paediatric rhabdomyosarcomas.

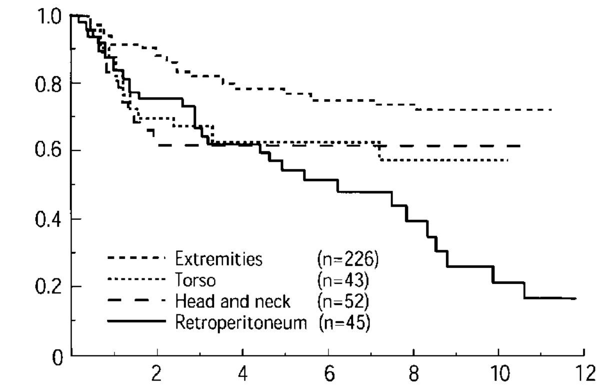

**Fig. 2.** *Site-specific survival in patients with soft tissue sarcoma. Overall survival from the time of diagnosis is shown for patients treated with curative intent at The Princess Margaret Hospital for soft tissue sarcoma of the extremity, trunk, or head and neck (1980–1988), and retroperitoneum (1975–1988) (modified from References 1 and 19, with permission).*

approach in improving long-term disease control is, however, difficult to prove.

Post-operative radiotherapy is frequently given for RPS, but there are significant barriers to its efficacy. Accurate determination of the radiation treatment volume may be compromised by incomplete documentation of the extent of disease. In addition, the radiation dose is usually limited to less than 50 Gy both by sensitive critical structures in the field and by the size of the treatment volume needed.[10] Not surprisingly, there is no clear evidence that post-operative radiation significantly reduces the risk of local recurrence after a grossly complete resection. However, post-operative radiation may delay the time to recurrence (Fig. 3), suggesting that external beam radiation might be effective if an adequate dose could be given to the tissues at risk. This has prompted an interest in other strategies for adjuvant radiation delivery, including pre-operative external beam radiation, intraoperative radiation therapy, and post-operative brachytherapy. The addition of systemic chemotherapy is another strategy undergoing evaluation in some centres.

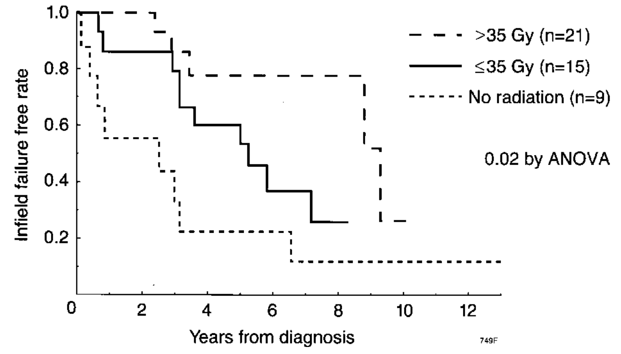

**Fig. 3.** *Effect of post-operative radiation on time to recurrence in RPS. The proportion of patients remaining free of infield recurrence from the time of diagnosis is shown for 45 patients with RPS treated with grossly complete resection and post-operative external beam radiation. Patients are grouped according to the dose of radiation given, as indicated (from Reference 19, reproduced with permission).*

Our understanding of RPS is based largely on retrospective single-institutional experiences, which usually report on small numbers of patients who were treated non-uniformly over several decades. Limited patient numbers and great variability in extent of local disease make it difficult to conduct meaningful randomized clinical trials, and even non-randomized prospective trials are rare. In this review, we briefly describe the presentation, natural history, diagnosis, and investigation of patients with RPS, and focus on the results of treatment as documented in the modern literature. New treatment strategies are presented and the need for centralized, multidisciplinary care as well as for multicentre collaboration in conducting prospective trials is emphasized.

### Presentation and natural history

According to population-based data from the Surveillance, Epidemiology and End Results (SEER) Program,[11] the age-adjusted annual incidence of all soft tissue sarcomas (excluding epidemic Kaposi's) is about 5 per 100,000 in the United States. According to the SEER data, RPS accounted for 10% of sarcomas arising in all sites (1602 of 16,067 cases, Kaposi's sarcoma excluded). RPS had an equal incidence in males and females, and a median age at presentation of 61.5 years.[11] Most large case series of RPS concur with these demographic data. There are few recognized aetiologic factors for soft tissue sarcoma. These include the development of radiation-induced tumours, and sarcomas arising in patients with known genetic mutations, such as malignant peripheral nerve sheath tumours in neurofibromatosis, and various types of soft tissue sarcoma in the Li–Fraumeni syndrome.[12]

RPS usually arise from the connective tissues posterior to the posterior peritoneum, and uncommonly from specific retroperitoneal tissues such as the kidney, inferior vena cava, spinal nerve roots or the aorta.[13,14] RPS often grow silently to a very large size before diagnosis. Patients typically present with chronic non-specific complaints related to tumour compression rather than infiltration,[15] including abdominal distension and pressure, early satiety and anorexia, changes in bowel or bladder habit, and peripheral oedema. Not infrequently, the diagnosis is made on an incidentally found asymptomatic mass.[16] Cross-sectional imaging may demonstrate a massive tumour markedly displacing intra-abdominal organs (Fig. 4), and the lack of associated symptoms implies that the patient has adapted to indolent tumour growth. Patients with high-grade tumors may present with a rapidly growing abdominal mass, pain or more severe constitutional symptoms, particularly in the presence of metastatic spread. This constellation of symptoms may also represent the development of de-differentiated areas in an otherwise apparently well-differentiated liposarcoma.[17]

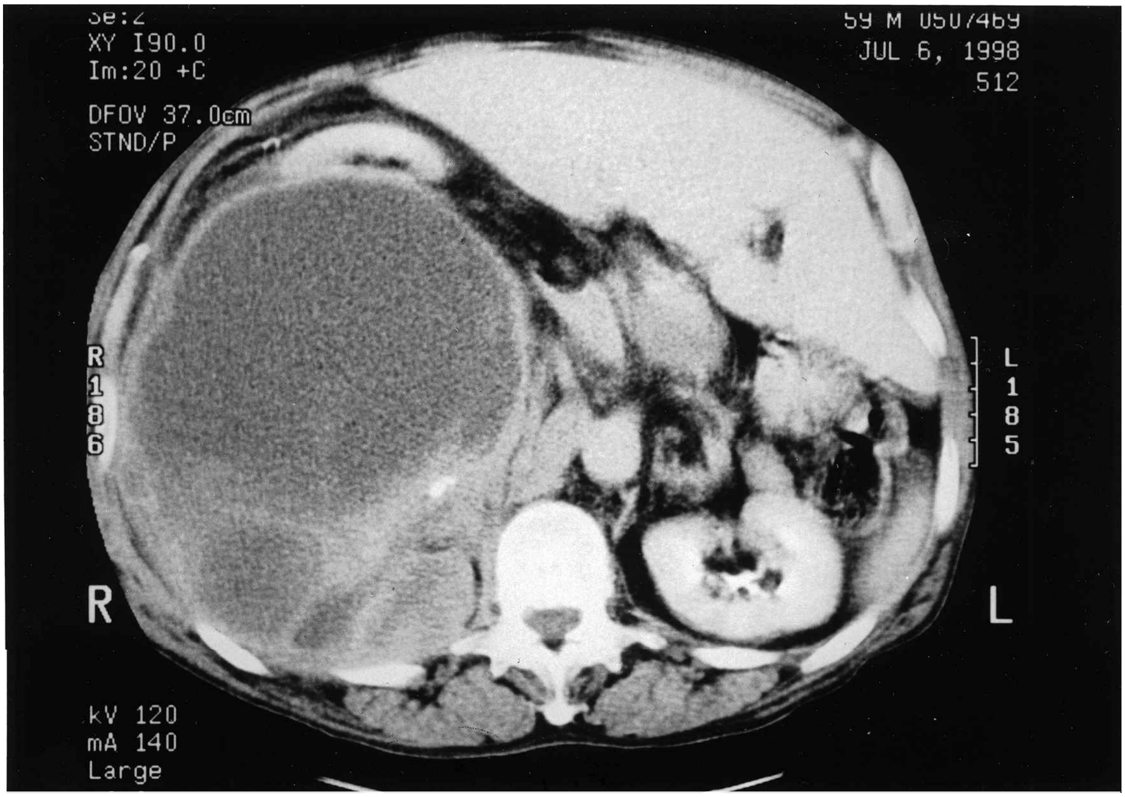

**Fig. 4.** *Displacement of viscera by tumour pre-operatively. A patient with a large right-sided RPS that displaces the liver, bowel, and the ipsilateral kidney.*

Between 10 and 20% of patients with RPS are found to have distant metastases at the time of initial presentation. Of those who present with non-metastatic disease and undergo curative therapy, about 25% will develop metastases, most commonly in the liver and lungs.[4,5] This metastatic rate is unexpectedly low considering the high proportion of patients who present with very large tumours and who do not achieve local control. Whether RPS is associated with an inherently limited metastatic potential or whether the incidence of metastases is under-reported is not clear. Local and systemic recurrences can develop late: in one large series, 14% of all failures occurred 5–12 years after diagnosis.[18] This highlights the importance of long-term follow-up after curative therapy for these patients.

The various histologic subtypes of adult RPS are presented in Table 2. As in most recent series, the three most common histologies (liposarcoma, leiomyosarcoma and malignant fibrous histiocytoma) accounted for about 70% of the total RPS reported in the SEER study. Overall, 36–50% of RPS are scored as low grade, in contrast to 19–26% of extremity and truncal sarcomas.[1,3–5,7,19–21] This could explain the lower incidence and/or delayed appearance of metastases in patients with RPS.

### Diagnosis and pretreatment evaluation

Computerized axial tomography (CT) is particularly useful in the diagnosis, staging and pretreatment planning of RPS.[22] Encasement of major vessels and involvement of adjacent organs, as well as identification of lung and liver metastases, are of particular interest. Ultrasound and magnetic resonance imaging (MRI) are useful complements to CT in characterizing liver lesions, while MRI gives more detailed information about neurovascular and muscular involvement.

Characteristics of retroperitoneal tumours that predict for malignancy on CT are size > 5.5 cm, absence of calcifications, irregular margins, and cystic degeneration or necrosis.[23] Fatty tumours are readily identified on CT by their low attenuation, and

**Table 2.** *Frequency of histological subtypes of retroperitoneal sarcoma**

| Histology | Frequency (%) |
|---|---|
| Liposarcoma | 30.0 |
| Leiomyosarcoma | 26.7 |
| Malignant fibrous histiocytoma | 14.9 |
| Sarcoma not otherwise specified | 9.4 |
| Fibrosarcoma | 4.5 |
| Malignant neurilemmoma | 2.6 |
| Neurofibrosarcoma | 2.1 |
| Malignant mesenchymoma | 2.0 |
| Rhabdomyosarcoma (excluding embryonal) | 1.9 |
| Malignant hemangiopericytoma | 1.2 |
| Hemangiosarcoma | 0.7 |
| Myxosarcoma | 0.3 |
| Malignant hemangioendothelioma | 0.09 |
| Epithelioid sarcoma | 0.09 |

* From SEER.[11]

the presence of high attenuation areas in a fatty tumour may indicate that de-differentiation has taken place. CT-Guided percutaneous needle biopsy of these areas may provide confirmation.

The pretreatment evaluation of a patient with possible RPS should include: (i) a general medical assessment; (ii) biopsy with histologic classification and grading; (iii) a thorough evaluation of local tumour extent; and (iv) a metastatic search, with particular attention to the lungs and liver. A radio-isotope differential renal function scan is useful for evaluating the function of the contra-lateral kidney when *en bloc* resection of the homolateral kidney is contemplated.

The amount of tissue obtainable from an open biopsy usually provides the pathologist the best opportunity to fully classify and grade the sarcoma prior to treatment. However, an open biopsy is potentially morbid, and may delay treatment. In addition, the peritoneal cavity may become contaminated with tumour. CT-guided needle biopsy carries a lower potential for morbidity and, when performed with the posterior approach, has a low risk of contaminating uninvolved areas. In our centre, we have found CT-guided core needle biopsy to be safe and efficacious in making a tissue diagnosis of retroperitoneal tumours. The interpretation of small tissue specimens can be difficult, however, and we recommend that both the biopsy procedure and the pathological evaluation be carried out in centres with experience in managing soft tissue sarcomas. Ideally, pathological evaluation of the biopsy specimen should not only confirm the diagnosis of sarcoma, but also provide the histological subtype and grade. In practice, we accept the diagnosis of sarcoma NOS (not otherwise specified) as sufficient to plan and undertake therapy. If the diagnosis cannot be made despite a second CT-guided biopsy, an open biopsy is usually required. This biopsy should be planned with the eventual resection in mind, and executed so as to minimize contamination of uninvolved tissues.

### Outcome: patterns of failure and prognostic variables

Table 1 presents patient outcome as quoted in all individual series of 30 or more cases of adult RPS published in English since 1980 ($n = 31$). Series that do not separate RPS from intra-abdominal sarcomas or other miscellaneous tumours have been excluded. Where the same centre has published sequential series that include patients all included in a previous series, only the most recent qualifying publication is included.

As shown in Table 1, the overall survival reported for patients with RPS varies from 11 to 63% at 5 years, and from 10 to 50% at 10 years following initial presentation. The wide spectrum of survival rates is at least partially due to differences in the patient population making up the denominator in each study. Failure to control local disease is a contributing factor towards death in almost 90% of patients, and is due either to an inability to completely resect the primary or to local relapse following total gross resection. A variety of potential prognostic variables have been investigated.

### *Complete resection*

The only treatment factor that consistently predicts for improved survival is a grossly complete tumor resection, and complete resection rates are typically reported as under 70%. Survival rates at 5 and 10 years after complete resection are approximately 60 and 25%, respectively. Recent improvements in preoperative assessment, and a more aggressive surgical approach to include resection of involved viscera and other structures, have improved complete resection rates to 80–95% in some series. Karakousis *et al.*[21] report a complete gross resection rate of 96%, and associated 5- and 10-year survival rates of 63 and 46%. Some of this improvement is a result of better pretreatment selection of patients for surgical exploration, but it is likely that there has also been a real increase in complete resection rates.

### *Size*

Tumour size has generally not been shown to influence survival, local failure or distant failure rates. This reflects the fact that RPS are almost always very large at presentation, most being larger than 10 cm in diameter,[4,24,25] so that no series includes a sufficient number of smaller tumours to demonstrate an effect on outcome.

### *Grade*

High grade predicts for decreased survival in many series,[3,4,6–8,10,20,21,24,26–29] but not in others.[18,19,30,31] In one large series,[18] high grade predicted for increased metastatic failure 5 years or more after initial presentation, but not for an increased risk of mortality. The development of metastatic disease in long-term survivors of RPS may represent de-differentiation of low-grade disease after many years.

### *Histology*

Most series are too small to meaningfully evaluate the effect of histological subtype on prognosis. Some series have shown improved survival in patients with liposarcoma compared with the other histologies (univariate analysis only),[19,27] but not an improved local relapse free rate.[19] This may reflect the generally more indolent growth pattern of liposarcoma. In a large prospective series, Lewis *et al.*[4] recently reported no independent effect of histology on survival in a multivariate analysis, although liposarcoma histology predicted for an increased risk of local recurrence. It seems likely that the retroperitoneal site is itself the predominant determinant of outcome, rather than histological subtype.

### *Local failure*

Retreatment of local relapse is generally associated with poorer survival, even with aggressive resection. Karakousis *et al.*[21] reported 10-year survival rates of 57% for primary tumours versus 26% for locally recurrent tumours. Lewis *et al.*[4] reported 5-year disease-specific survival rates of 54 and 22% for patients presenting with primary disease and local recurrence, respectively. In the latter series, those who developed local relapse after complete resection of a primary tumour had a median survival of only 28 months. Wang *et al.*[32] reported a 12% survival rate at 96 months for patients with completely excised recurrent tumours, again indicating that the long-term salvage rate is quite low for recurrent RPS. Local control rates are similarly compromised in patients presenting with recurrent disease, due at least in part to lower complete resection rates, but probably also as a result of unfavourable biology.[21,33]

### *Adjuvant radiation*

In some series (retrospective, non-randomized), treatment with external beam radiation after complete resection has apparently been associated with improved outcome. One report showed an improvement in actuarial 10-year local relapse free survival from 35 to 55% in unirradiated versus radiated patients following complete resection.[18] In a small series, Tepper *et al.*[34] reported improved local control for patients who received more than 60 Gy compared with less than 50 Gy. Fein *et al.*[31] reported local control rates of 72% versus 38% in patients who received more versus less than 55 Gy; these rates were measured at 2 years post-treatment. Catton *et al.*[19] reported a similar differential in local control rates at 2 and 5 years for patients who received greater or less than 35 Gy following complete excision; by 10 years, however, the local relapse rates had reached equivalence. In a randomized trial designed to test the effect of additional intraoperative radiotherapy in patients who all received external beam postoperatively, Sindelar *et al.*[35] found that an intraoperative boost that brought the total dose to 60 Gy reduced the local relapse rate from 80 to 40% ($p < 0.05$) at 8 years. However, there was no difference in overall or disease-free survival rates between the two treatment arms.

Overall, the available evidence suggests that low-dose postoperative radiation is of little benefit in preventing local relapse, and that increasing the dose delays but does not prevent local recurrence in the majority of patients. Furthermore, post-operative radiation is clearly associated with significant risk of severe acute and late bowel toxicity.[10]

### *Adjuvant chemotherapy*

The evidence for the routine use of adjuvant chemotherapy in localized, resectable soft tissue sarcoma is controversial. Individual randomized trials have not shown a conclusive benefit, but a recent meta-analysis[36] of 1568 patients with localized, resectable soft tissue sarcomas at various sites indicated that doxorubicin-based chemotherapy was associated with a significant delay in both local and distant relapse. There was a trend towards an improvement in overall survival, but it did not reach statistical significance. Patients with RPS were not identified separately in the analysis. A small randomized trial of adjuvant chemotherapy for RPS[10] showed inferior survival and severe treatment toxicity for the patients receiving chemotherapy.

### **New treatment strategies**

The importance of achieving complete gross resection of RPS has been emphasized. At major sarcoma centres, the following strategies are currently employed to optimize complete resection rates: (i) detailed pre-operative assessment with the routine use of cross-sectional imaging and needle biopsy to improve patient selection; (ii) aggressive surgery to include *en bloc* resection of involved viscera and other expendable structures; and (iii) improved specialized post-operative care and rehabilitation. These tactics have resulted in reported resection rates as high as 95%, with low perioperative mortality. It is not altogether clear to what extent these high resection rates reflect improvements in pre-operative patient selection rather than in overall resectability. Over a recent 3-year period (1996–1998), 25 patients with RPS were referred to our centre with a tumour *in situ*. Five had metastases at presentation, three were considered unresectable after investigation, and two declined any therapy. The remainder underwent complete resection, for an overall resectability rate of 60%, and a total gross resection rate of 100% in those selected for operative exploration. Seventy-eight percent of the latter group required *en bloc* resection of adjacent viscera. There is probably a selection bias for adverse features in the patients referred to our centre. Nevertheless, our recent experience suggests that the current surgical approach has increased overall resection rates.

Post-operative radiation therapy is the standard adjuvant treatment for extremity sarcoma, but it is not effective in preventing local recurrence of RPS. Possible reasons for this include the use of inadequate radiation doses, inadequate treatment volumes, or both. The presence of adhesed small bowel in the tumour bed post-operatively impedes the delivery of a full radical dose (Fig. 5). Strategies to improve radiation

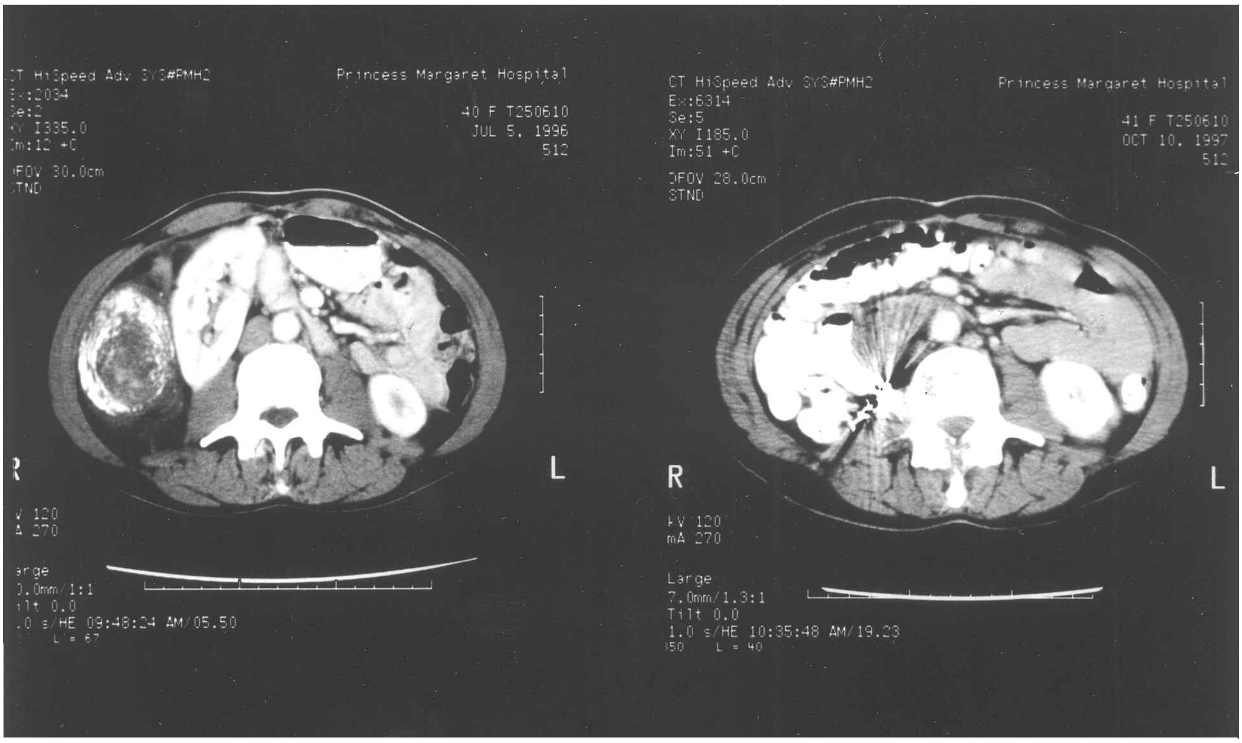

**Fig. 5.** *Comparison of the radiation field pre- and post-operatively. Pre- and post-operative CT scans of a patient with a right-sided RPS are shown in the left- and right-hand panels, respectively. The post-operative film demonstrates that the previously displaced bowel has fallen back into the tumour bed after resection of the tumour and right kidney.*

delivery and to escalate the total dose of radiation include pre-operative radiotherapy, intraoperative placement of tissue expanders to displace the small bowel out of the post-operative radiation field,[37] and use of brachyradiation or intraoperative electron beam techniques.[35,38–43] The latter strategies may be employed in conjunction with conventional external beam radiotherapy to escalate the dose to the tumour bed. Radiation targeted specifically at the tumour bed may be delivered intraoperatively (intraoperative radiation therapy (IORT)) with an electron beam directed through an appropriately positioned cone, or with a high dose rate brachytherapy applicator,[33] or it may be administered in the post-operative period with low dose rate or pulsed dose rate brachytherapy through intraoperatively placed catheters.[39]

The experience with IORT for RPS has been mixed. Gieschen *et al.*[40] recently reported very impressive local (91%) and distant (80%) 5-year control rates in 16 patients who received electron-beam IORT after pre-operative external beam therapy and complete gross resection with moderate morbidity. Using post-operative external beam radiation plus electron-beam IORT, Bussieres *et al.*[38] found a 2-year relapse free rate of 60%, with acute and late complication rates of 21 and 31% respectively. Alektiar *et al.*[33] reported the Memorial Sloan–Kettering experience with high dose rate IORT given with or without external beam therapy for 32 patients. In this recent series, the overall 5-year local control rate was 74% for patients presenting with primary disease and 54% for those with recurrent tumours. A 58% local control rate was found by Petersen *et al.*[44] in 87 patients treated at the Mayo clinic with electron-beam IORT and external beam therapy. Peripheral neuropathy and hydronephrosis appear to be significant sources of long-term morbidity following retroperitoneal IORT.[40,42,44]

A randomized study of post-operative radiotherapy with or without an intraoperative boost did not show a significant benefit for dose escalation in the post-operative setting.[35] This trial had insufficient power to detect small differences in outcome, but the intra-abdominal relapse rate was very high in both study arms. It is possible that some patients did not benefit from dose escalation because the treatment volumes were not adequate to cover all the areas at risk. This hypothesis is supported by the findings of Sugarbaker *et al.*[45] who reported the patterns of failure after surgery for RPS. Recurrences were identified as expected in the tumour bed and in sites of previous tumour involvement, but also in sites of surgical trauma and in nodules studding the peritoneal surface. The number of sites of relapse increased with the number of operations performed. This observation supports the theory that intra-abdominal tumour emboli are frequent at the time of primary surgery, and that these emboli are released at resection to become entrapped in fibrinous material along narrow resection margins and at other sites of surgical trauma. The complex cytokine and protease cascades involved in wound healing may further contribute to the establishment of tumour emboli diffusely in the peritoneal and retroperitoneal spaces.

Tumour resection may thus expand the areas at risk of failure well beyond the tumour bed. Accurate identification and effective treatment of the target volume with post-operative radiation is clearly problematic. Treating patients with the tumour *in situ* provides the major advantage of having cross-sectional imaging available to plan the radiation fields directly onto the tumour. In addition, the tumour mass acts as a tissue expander displacing sensitive structures out of the radiation field (Figs. 4 and 5), and very large volumes can be treated to 45 or 50 Gy with minimal acute toxicity.[39,43,46] Pre-operative external beam therapy may also decrease the risk of tumour implantation at resection by sterilizing the operative field of microscopic tumour emboli prior to resection.

Another potentially beneficial strategy is to escalate the dose of radiation delivered to the tumour bed. We have reported dose escalation to 70 Gy using pre-operative external beam radiation and post-operative pulsed dose rate brachytherapy in 13 completely resected patients. Severe duodenitis, the most frequent acute side effect, was seen in 35% of patients treated with this combined therapy regimen. The majority of patients responded to medical management and 86% were symptom free by 90 days after therapy.[39]

Based on the rationale already outlined and on the recent experience of our group and others, we feel that pre-operative radiation is the preferable method of delivering adjuvant external beam radiotherapy for RPS. It is better tolerated, it permits the radiation to be directed more precisely to the tissues at risk, and it may reduce the risk of tumour implantation at resection. It appears that dose escalation to the tumour bed with brachytherapy catheters or IORT may be given relatively safely following pre-operative radiation, but clinical trials with larger patient numbers and long-term follow-up are required to determine the true morbidity and whether this approach will in fact improve local control.

Pre-operative chemotherapy has been proposed as an adjunct to surgery and radiotherapy to improve resectability, and to reduce the risk of local and systemic relapse. Robertson *et al.*[47] reported a trial of pre-operative radiotherapy with the radiosensitizer iododeoxyuridine in 16 patients with locally advanced RPS. This combined therapy was well tolerated, and was associated with a complete resection rate of 50% and a 2-year local relapse-free rate of 46%. Sugarbaker[48] has proposed post-operative intraperitoneal adriamycin as a method of reducing local recurrence, and Eroğlu *et al.* recently reported initial favourable results with intraoperative hyperthermic total abdominal perfusion in 11 patients.[24] The sarcoma group at MD Anderson is investigating the use of an intensive pre-operative chemoradiation regimen with the goal of improving resectability, and reducing the risk of local and systemic failure.[61]

### Conclusion

Better patient selection and more aggressive *en bloc* tumour resection have resulted in improved complete resection rates for RPS at specialized centres. Nevertheless, the majority of patients managed by resection alone will experience local relapse. At the present time, most centres treat potentially curable RPS with combined therapy consisting of resection and irradiation. Post-operative external beam radiation is associated with significant morbidity and is not effective in preventing relapse, but may delay it. There are technical advantages to pre-operative radiation for both target delineation and sparing of normal tissue, but the effectiveness of pre-operative radiation has not been assessed in a randomized trial. Innovative strategies to escalate the radiation dose via an intra-operative or post-operative boost have been tested in small numbers of patients at various individual centres with varying results. The role of adjuvant chemotherapy in RPS is under investigation at a few centres, but currently remains undefined.

Our present policy is to encourage pre-operative referral and to enter eligible patients into a phase II trial of pre-operative radiation with a pulsed dose rate brachytherapy boost to the tumour bed after complete resection. The use of adjuvant radiotherapy for patients who present after complete resection is controversial. Our policy is to selectively offer post-operative radiotherapy only after an evaluation of the treatment risks, the likelihood of adequate tumour coverage, the risk of recurrence, and the likelihood of salvaging a relapse if the initial treatment fails. It is usually impossible to identify or cover an adequate treatment volume after an unplanned intralesional excision. The patient who has had a planned, *en bloc* excision and a careful pathological assessment may have well-defined areas of microscopic residual disease that are marked with surgical clips. Small bowel in the tumour bed usually limits the dose actually delivered to less than 50 Gy. Overall, we have concluded that post-operative adjuvant radiation is unlikely to be of significant benefit to patients with RPS. Patients with a primary tumour that cannot be completely resected are managed with palliative intent, using chemotherapy, radiation therapy and operative intervention as deemed appropriate. The same approach is generally also adopted in patients with metastatic disease.

We recommend that patients in whom the diagnosis of RPS is suspected should be referred to a multidisciplinary sarcoma unit before resection is attempted. Centralized multidisciplinary care allows the development and concentration of the expertise necessary to manage RPS appropriately, and ensures optimal pre-treatment investigation. In addition, referral to a specialized sarcoma centre increases the number of patients available for accrual into clinical trials. RPS is a rare disease. At initial presentation, up to 40% of patients do not qualify for treatment with curative intent, further limiting the pool of patients who may qualify for studies of adjuvant therapy. There is clearly a need for multicentre collaboration to accrue adequate numbers of patients to clinical trials.

### References

1 Levay J, O'Sullivan B, Catton CN, Bell R, Fornasier V, Cummings B, Hao Y, Warr D, Quirt I. Outcome and prognostic factors in soft tissue sarcoma in the adult. *Int J Radiat Oncol Biol Phys* 1993; 27:1091–9.

2 Wilson AN, Davis A, Bell RS, O'Sullivan B, Catton C, Madadi F, Kandel R, Fornasier VL. Local control of soft tissue sarcoma of the extremity: the experience of a multidisciplinary sarcoma group with definitive surgery and radiotherapy. *Eur J Cancer* 1994; 30A:746–51.

3 Alvarenga JC, Ball ABS, Fisher C, Fryatt I, Jones L, Thomas JM. Limitations of surgery in the treatment of retroperitoneal sarcoma. *Br J Surg* 1991; 78:912–6.

4 Lewis JJ, Leung D, Woodruff JM, Brennan MF. Retroperitoneal soft tissue sarcoma. Analysis of 500 patients treated and followed at a single institution. *Ann Surg* 1998; 228:355–65.

5 Storm FK, Eilber FR, Mirra J, Morton DL. Retroperitoneal sarcomas: a reappraisal of treatment. *J Surg Oncol* 1981; 17:1–7.

6 Bevilacqua RG, Rogatko A, Hajdu SI, Brennan MF. Prognostic factors in primary retroperitoneal soft-tissue sarcomas. *Arch Surg* 1991; 26:328–34.

7 Dalton R, Donohue JH, Mucha P Jr, van Heerden JA, Reiman HM, Chen S. Management of retroperitoneal sarcomas. *Surgery* 1989; 106:725–33.

8 Jaques DP, Coit DG, Hajdu SI, Brennan MF. Management of primary and recurrent soft-tissue sarcoma of the retroperitoneum. *Ann Surg* 1990; 212:51–9.

9 McGrath PC, Neifeld JP, Lawrence W, *et al.* Improved survival following complete excision of retroperitoneal sarcomas. *Ann Surg* 1984; 200:200–4.

10 Glenn J, Sindelar WF, Kinsella T, *et al.* Results of multimodality therapy of resectable soft-tissue sarcomas of the retroperitoneum. *Surgery* 1985; 97:316–24.

11 Mack TM. Sarcomas and other malignancies of soft tissue, retroperitoneum, peritoneum, pleura, heart, mediastinum, and spleen. *Cancer* 1995; 75(suppl 1):211–44.

12 Strong LC, Williams WR, Tainsky MA. The Li–Fraumeni syndrome: from clinical epidemiology to molecular genetics. *Am J Epidemiol* 1992; 135:190–9.

13 Compagnoni G, Matsumura JS, Nemcek A, McCarthy WJ. Sarcoma arising from an abdominal aortic aneurysm. *Ann Vasc Surg* 1997; 11:183–5.

14 Mingoli A, Sapienza P, Cavallaro A, *et al.* The effect of extended caval resection in the treatment of inferior vena cava leiomyosarcoma. *Anticancer Res* 1997; 17:3877–81.

15 Ziran BH, Makley JT, Carter JR. Primary retroperitoneal sarcomas: common symptoms, common diagnoses, uncommon disease. *Clin Orthop* 1996; 331:277–82.

16 Bolin TE, Bolin SG, Wetterfors J. Retroperitoneal sarcomas: an analysis of 32 cases. *Acta Chir Scand* 1988; 154:3–20.

17 Hendricks WH, Chu YC, Goldblum JR, Weiss SW. Dedifferentiated liposarcoma: a clinicopathological analysis of 155 cases with a proposal for an expanded definition of dedifferentiation. *Am J Surg Pathol* 1997; 21:271–81.

18 Heslin MJ, Lewis JJ, Nadler E, Newman E, Woodruft JM, Casper ES, Leung D, Brennan MF. Prognostic factors associated with long-term survival for retroperitoneal sarcomas: implications for management. *J Clin Oncol* 1997; 15:2832–39.

19 Catton CN, O'Sullivan B, Kotwall C, Cummings BJ, Hao Y, Fornasier VL. Outcome and prognosis in retroperitoneal soft tissue sarcoma. *Int J Radiat Oncol Biol Phys* 1994; 29:1005–10.

20 Cody HS, Turnbull AD, Fortner JG, Hajdu SI. The continuing challenge of retroperitoneal sarcomas. *Cancer* 1981; 47:2147–52.

21 Karakousis CP, Gertsenbluth R, Kontzoglou K, Driscoll DL. Retroperitoneal sarcomas and their management. *Arch Surg* 1995; 130:1104–9.

22 Munk PL, Lee MJ, Poon Py, Goddard KJ, Knowling MA, Hassell PR. Computed tomography of retroperitoneal and meserentic sarcomas: a pictoral essay. *Can Assoc Radiol J* 1996; 47:335–41.

23 Nakashima J, Ueno M, Nakamura K, *et al.* Differential diagnosis of primary benign and malignant retroperitoneal tumors. *Int J Urol* 1997; 4:441–6.

24 Eroğlu A, Kocaoğlu H, Demirci S, Akgül A. Retroperitoneal soft tissue sarcoma: effect of hyperthermic total abdominal perfusion. *Tumori* 1999; 85:259–64.

25 Wang T-Y, Lo S-S, Wu C-W, Lui W-Y. Surgical management of primary retroperitoneal sarcoma. *Chin Med J (Taipei)* 1996; 58:177–82.

26 Jenkins MP, Alvaranga JC, Thomas JM. The management of retroperitoneal soft tissue sarcomas. *Eur J Cancer* 1996; 32A:622–6.

27 Kilkenny JW III, Bland KI, Copeland EM III. Retroperitoneal sarcoma: the University of Florida experience. *J Am Coll Surg* 1996; 182:329–39.

28 Singer S, Corson JM, Demetri GD, Healey EA, Marcus K, Eberlein TJ. Prognostic factors predictive of survival for truncal and retroperitoneal soft-tissue sarcoma. *Ann Surg* 1995; 221:185–95.

29 Stockle E, Sastre FX, Bonvalot G, *et al.* Prognostic factors in retroperitoneal sarcoma. Analysis of a series of 165 patients of the French Cancer Center Federation (FNCLCC) sarcoma group [abstract]. *Sarcoma* 1999; 3:47.

30 Elgar F, Goldblum JR. Well-differentiated liposarcoma of the retroperitoneum: a clinicopathologic analysis of 20 cases, with particular reference to the extent of low-grade dedifferentiation. *Mod Pathol* 1997; 10:113–20.

31 Fein DA, Corn BW, Lanciano RM, Herbert SH, Hoffman JP, Coia LR. Management of retroperitoneal sarcomas: does close escalation impact on locoregional control? *Int J Radiat Oncol Biol Phys* 1995; 31:129–34.

32 Wang YN, Zhu WQ, Shen ZZ, LI S, Liu SY. Treatment of locally recurrent soft tissue sarcomas of the retroperitoneum: report of 30 cases. *J Surg Oncol* 1994; 56:213–6.

33 Alektiar KM, Hu K, Anderson L, Brennan MF, Harrison LB. High-dose-rate intraoperative radiation therapy (HDR-IORT) for retroperitoneal sarcomas. *Int J Radiat Oncol Biol Phys* 2000; 47:157–63.

34 Tepper JE, Suit HD, Wood WC, Proppe KH, Harmon D, McNulty P. Radiation therapy of retroperitoneal soft tissue sarcomas. *Int J Radiat Oncol Biol Phys* 1984; 10:825–30.

35 Sindelar WF, Kinsella TJ, Chen PW, DeLaney TF, Tepper JE, Rosenberg SA, Glatstein E. Intraoperative radiotherapy in retroperitoneal sarcomas — final results of a prospective, randomized, clinical trial. *Arch Surg* 1993; 128:402–10.

36 Sarcoma Meta-Analysis Collaboration. Adjuvant chemotherapy for localized resectable soft-tissue sarcoma of adults: meta-analysis of individual data. *Lancet* 1997; 350:1647–54.

37 Ball A, Cassoni A, Watkins R, Thomas J. Silicone implant to prevent visceral damage during adjuvant radiotherapy for retroperitoneal sarcoma. *Br J Radiol* 1990; 63:346–8.
38 Bussieres E, Stockle EP, Richaud PM, *et al.* Retroperitoneal soft tissue sarcomas: a pilot study of intraoperative radiation therapy. *J Surg Oncol* 1996; 62:49–56.
39 Catton CN, Swallow CJ, O'Sullivan B, Couture J, Kandel RA. A pilot study of external beam radiotherapy and pulsed dose rate brachytherapy for resectable retroperitoneal sarcomas [abstract]. *Radiother Oncol* 1998; 47(suppl 1):S30.
40 Gieschen HL, Willett CG, Spiro IJ, Suit HJ, Rattner DW, Ott MJ. Long-term results of intraoperative electron beam radiotherapy for primary and recurrent retroperitoneal sarcoma [abstract]. *Int J Rad Oncol Biol Phys* 1998; 42(suppl):192.
41 Kinsella TJ, Sindelar WF, Lack E, Glatstein E, Rosenberg SA. Preliminary results of a randomized study of adjuvant radiation therapy in resectable adult retroperitoneal soft tissue sarcomas. *J Clin Oncol* 1988; 6:18–25.
42 Sindelar WF, Hoekstra HJ, Kinsella TJ. Surgical approaches and techniques in intraoperative radiotherapy for intra-abdominal, retroperitoneal, and pelvic neoplasms. *Surgery* 1988; 103:247–56.
43 Willett CG, Suit HD, Tepper JE, *et al.* Intraoperative electron beam radiation therapy for retroperitoneal soft tissue sarcoma. *Cancer* 1991; 68:278–83.
44 Petersen IA, Haddock MG, Donohue JH, Nagomery DM, Gunderson LL, Grill JP. Use of intraoperative electron beam radiation therapy in the management of soft tissue sarcomas. *Int J Radiat Oncol Biol Phys* (in press).
45 Sugarbaker PH. Patterns of spread of recurrent intraabdominal sarcoma. *Cancer Treat Res* 1996; 82:65–77.
46 Gronchi A, Azzarelli A, Zanini M, *et al.* Preoperative tailored radiation therapy for advanced retroperitoneal soft tissue sarcomas. A feasibility study [abstract]. *Sarcoma* 1999; 3:44.
47 Robertson JM, Sondak VK, Weiss SA, Sussman JJ, Chang AE, Lawrence TS. Preoperative radiation therapy and iododeoxyuridine for large retroperitoneal sarcomas. *Int J Radiat Oncol Biol Phys* 1995; 31:87–92.
48 Sugarbaker PH. Early postoperative intraperitoneal adriamycin as an adjuvant treatment for visceral and retroperitoneal sarcomas. *Cancer Treat Res* 1996; 81:7–14.
49 Herman K, Gruchala A, Niezabitowski A, Glinski B, Lakowska B. Prognostic factors in retroperitoneal sarcomas: ploidy of DNA as a predictor of clinical outcome. *J Surg Oncol* 1999; 71:32–5.
50 Malerba M, Doglietto GB, Carriero C, *et al.* Primary retroperitoneal soft tissue sarcomas: results of aggressive surgical treatment. *World J Surg* 1999; 23(7):670–5.
51 van Doorn RC, Gallee PW, Hart AA, *et al.* Resectable retroperitoneal soft tissue sarcomas. *Cancer* 1994; 73:637–42.
52 Shiloni E, Szold A, White DE, Freund HR. High-grade retroperitoneal sarcomas: role of an aggressive palliative approach. *J Surg Oncol* 1993; 53:197–203.
53 Zornig C, Weh HJ, Krull A, *et al.* Retroperitoneal sarcoma in a series of 51 adults. *Eur J Surg Oncol* 1992; 18:475–80.
54 Pinson CW, ReMine SG, Fletcher WS, Braasch JW. Long-term results with primary retroperitoneal tumors. *Arch Surg* 1989; 124:1168–73.
55 Salvadori B, Cusumano F, Delledonne V, De Lellis R, Conti R. Surgical treatment of 43 retroperitoneal sarcomas. *Eur J Surg Oncol* 1986; 12:29–33.
56 Karakousis CP, Velez AF, Emrich LJ. Management of retroperitoneal sarcomas and patient survival. *Am J Surg* 1985; 150:376–80.
57 Wist E, Solheim OP, Jakobsen AB, Blom P. Primary retroperitoneal sarcomas. A review of 36 cases. *Acta Radiol Oncol* 1985; 24:305–10.
58 Shmookler BM, Lauer DH. Retroperitoneal leiomyosarcoma. A clinicopathologic analysis of 36 cases. *Am J Surg Pathol* 1983; 7:269–80.
59 Stower MJ, Hardcastle JD. Malignant retroperitoneal sarcoma: a review of 32 cases. *Clin Oncol* 1982; 8:257–63.
60 Fortner JG, Martin S, Hajdu S, Turnbull A. Primary sarcoma of the retroperitoneum. *Semin Oncol* 1981; 8:180–4.
61 Pisters PWT, Patel SR, Crane C, Feig BW, Hunt KK, Burgess MA, Papadopoulos NE, Plager C, Benjamin RS, Pollock RE, Janjan NE. Phase I trial of preoperative doxorubicin-based concurrent chemoradiation and electron-beam intraoperative radiation therapy (IORT) for resectable retroperitoneal sarcomas (RPS) [abstract]. *Proc Annu Meet Am Soc Clin Oncol* 2000; 19:A2199.
